# Human Dental Pulp Stem Cells Modulate Acute Inflammation Kinetics in the AIRmax Murine Model by Sustained TNF-Alpha Suppression and Transient Homing

**DOI:** 10.3390/cells15020189

**Published:** 2026-01-20

**Authors:** Bruna de Oliveira Policiquio, Vivian Gonzaga Fonseca, Geovanna Santos Costa, Jean Gabriel de Souza, Olga Celia Martinez Ibañez, Orlando Garcia Ribeiro, Irina Kerkis

**Affiliations:** 1 Genetics Laboratory, Butantan Institute, Sao Paulo CEP 05503-900, SP, Brazil; bruna.policiquio@butantan.gov.br (B.d.O.P.); vivian.gonzaga@butantan.gov.br (V.G.F.); geovannastcosta@gmail.com (G.S.C.); 2 Graduate Program in Structural and Functional Biology at UNIFESP, Federal University of Sao Paulo (UNIFESP), Sao Paulo CEP 04023-062, SP, Brazil; 3 Cellavita Pesquisas Científicas Ltd., Valinhos CEP 13278-381, SP, Brazil; 4 Centre of Excellence in NewTarget Discovery (CENTD), Butantan Institute, Sao Paulo CEP 05503-900, SP, Brazil; jean.souza@butantan.gov.br; 5 Immunogenetics Laboratory, Butantan Institute, Sao Paulo CEP 05503-900, SP, Brazil; olga.ibanez@butantan.gov.br (O.C.M.I.); orlando.ribeiro@butantan.gov.br (O.G.R.)

**Keywords:** mesenchymal stem cells (MSC), human dental pulp stem cells (hDPSCs), acute inflammatory response (AIR)

## Abstract

Mesenchymal stem cells (MSCs) are multipotent adult cells that are highly valued for their immunomodulatory potential and intrinsic ability to home to inflamed sites. This study specifically utilized human dental pulp stem cells (hDPSCs), a unique MSC subtype derived from the neural crest, due to their reported superior anti-inflammatory capacity. To rigorously test their efficacy, we employed the AIRmax murine model, which exhibits a genetically determined high-inflammatory phenotype. Acute inflammation was induced by subcutaneous injection of the polyacrylamide suspension Biogel P-100. Two hours post-induction, AIRmax mice were treated intravenously with hDPSCs. Our results demonstrate that hDPSC treatment produced significant anti-inflammatory effects evident at 24 h. The treated group showed a pronounced reduction in leukocyte migration and decreased protein extravasation in the inflammatory exudate. Crucially, hDPSCs also modulated molecular mediators, significantly decreasing the pro-inflammatory cytokine TNF-alpha and reactive oxygen species (ROS) production. Furthermore, while hDPSCs efficiently and rapidly homed to the inflammation site within 2 h, their maximal therapeutic benefits only manifested after 24 h. This suggests that their robust capacity to modulate acute inflammatory responses relies not only on rapid migration but also on a paracrine “hit-and-run” mechanism that suppresses cellular infiltration and oxidative stress over time. This study reinforces the potential of hDPSCs as a powerful, multi-target therapeutic agent for inflammatory conditions, supporting further investigation into their precise mechanisms and clinical application.

## 1. Introduction

Mesenchymal stem cells (MSCs) are remarkable adult, multipotent cells. They possess the vital capacity for self-renewal and can differentiate into all three primary cell types of the mesoderm [1]. While we do not fully understand how MSCs achieve their therapeutic effects, a major mechanism appears to be their ability to seek out and home in on pro-inflammatory sites, guided by chemokine signaling [2,3,4,5].

Once at the site of inflammation, MSCs act as sophisticated biological regulators. They sense the inflammatory environment and respond by releasing a complex cocktail of cytokines and chemokines, effectively helping to modulate the immune response [6,7]. This leads to a beneficial shift: a reduced level of pro-inflammatory cytokines and increased production of anti-inflammatory cytokines [8,9].

Furthermore, MSCs exert powerful control over the immune system by directly interacting with various immune cells, including T lymphocytes, B lymphocytes, natural killer (NK) cells, and antigen-presenting cells (APCs). Crucially, they suppress the activation and proliferation of T and B cells and reduce antibody production. Their immunosuppressive capabilities are particularly evident against T cells, NK cells, and APCs. They achieve this broad regulatory effect by expressing key mediators such as IL-6, IL-10, prostaglandin E2, nitric oxide, transforming growth factor β1 (TGFβ1), and hepatocyte growth factor [10,11,12,13,14].

This ability to migrate to injury sites—a process called homing, which responds to chemokine signals—and resolve inflammation by secreting paracrine factors that promote tissue regeneration and restore homeostasis makes MSCs highly valuable therapeutic candidates, especially for inflammatory conditions [15]. Given that the regenerative success of MSC-based therapies is intimately linked to their ability to promote immunomodulation [16,17], their potential for advanced cell treatments is widely recognized and extensively investigated [18].

While many sources of MSCs exist, those derived from primary (deciduous) teeth have garnered significant attention. Their key advantages are clear: they can be harvested through a relatively simple, minimally invasive extraction with low patient morbidity, and the pulp tissue provides an efficient yield of stem cells, making them ideal for tissue reconstruction applications [19].

Interestingly, dental pulp originates from the neural crest during embryonic development, giving its cell population a highly migratory and developmental potential—a profile like embryonic stem cells (ESCs) [20,21]. Building on this, Kerkis et al. (2006) [20] successfully isolated multipotent cells from the pulp tissue using an explant technique, under conditions like those used for ESC isolation. They yielded cells with an intermediate profile between ESCs and adult stem cells (ASCs) [22], which they termed human dental pulp stem cells (hDPSCs) [19]. At early passages, hDPSCs exhibit an immunophenotypic profile characteristic of ESCs (positive for Oct-4, Nanog, SSEA-3 and 4, TRA-1-60, and TRA-1-81), but they also express standard MSC markers (SH2, SH3, SH4, CD31, and CD13) observed in approximately 97% of cells [19,23].

The discovery and efficiency of hDPSCs mark a significant leap forward in regenerative medicine, offering new possibilities for the isolation of stem cells with high differentiation and self-renewal potential. Primary teeth offer an advantageous source of easily accessible cells, harvested with minimal discomfort to the patient. Therefore, these cells have become an attractive source for regenerative medicine [19].

Given what has been said, hDPSCs are a type of MSCs obtained from the dental pulp of deciduous teeth. This superior regulatory ability, coupled with their high proliferative potential in vitro, makes hDPSCs exceptionally suitable for scalable therapeutic applications [19,20]. In this regard, studies show that hDPSCs, obtained from the dental pulp of primary teeth of children aged 6–10 years, have an immunomodulatory potential approximately three times greater than that of other MSCs. This characteristic, combined with their high in vitro proliferative potential, allows these cells to be expanded on a scalable level for therapeutic purposes [20,24]. Thus, based on previous knowledge about the promising paracrine capacity of MSCs in inflammatory environments, the potential of these cells to act as immunomodulatory and anti-inflammatory agents was recognized.

The Immunogenetics Laboratory at the Butantan Institute developed two genetically heterogeneous mouse lines, AIRmax and AIRmin, to study the acute inflammatory response (AIR) and its genetic regulation. These mouse models were created by crossing eight isogenic strains: A/J, BALB/c, C57BL/6J, CBA/J, DBA/2J, P/J, SJL/J, and SWR/J [25].

The selection process was based on individual phenotypic characteristics of animals from the F0 population. Animals with high or low acute inflammatory responsiveness were selected, as determined primarily by the number of infiltrated leukocytes and the amount of extravasated proteins after injection with Biogel P100, a polyacrylamide gel and a non-immunogenic phlogistic agent. Mating was performed consecutively for several generations until the high- and low-responsive lines exhibited maximally separate phenotypes, designated AIRmax and AIRmin [25].

The AIRmax and AIRmin murine strains serve as an invaluable tool for studying inflammatory processes due to their genetically determined, contrasting inflammatory phenotypes. The AIRmax phenotype exhibits a significantly higher inflammatory response, notably demonstrating approximately twenty times higher leukocyte migration to the site of inflammation compared to the AIRmin mice [25,26,27,28,29].

The AIRmax model is uniquely suited for assessing MSCs’ efficacy because its robust response effectively simulates intense acute inflammation, characterized by high levels of leukocyte recruitment, inflammatory mediator secretion, and increased vascular permeability [25,26]. This environment provides an ideal and controlled platform for the following: observing MSC interactions with inflammatory mediators and host immune cells; investigating the immunomodulatory mechanisms of hDPSCs; and evaluating their potential as a therapeutic approach for severe acute inflammation [30,31,32].

Therefore, our study directly seeks to explore the therapeutic potential of hDPSCs in modulating this acute inflammation using the AIRmax model. Crucially, the hDPSCs utilized in this investigation were manufactured under good manufacturing practice (GMP) conditions, which significantly reinforces the clinical relevance and translational potential of our anti-inflammatory findings [33,34].

## 2. Materials and Methods

### 2.1. Human Dental Pulp Stem Cells (hDPSCs)

The hDPSCs used in this research were provided by Cellavita Pesquisas Científicas Ltd. (Valinhos, Brazil), a collaborative company of the Butantan Institute located in Valinhos, SP. Dental pulp stem cells were from the pulp of deciduous teeth donated by healthy individuals aged 5 to 12 years. The procedure was approved by an ethics committee (approval number 52375916.1.0000.5412), and written informed consent was obtained from both donors and their legal guardians for the use of their teeth in clinical research.

These cells were grown (expanded) in 75 cm^2^ polystyrene culture flasks under strict GMP conditions, adhering to Cellavita’s established standard operating procedures. The final product, marketed as NestaCell^®^, was transported to the Laboratory of Genetics of the Butantan Institute (São Paulo, Brazil) in a cryopreserved state at −80 °C. These hDPSCs were grown under GMP conditions, confirming their suitability for use in clinical studies [33,34].

### 2.2. AIRmax Model

The AIRmax mice originated from the animal colony of the laboratory of immunogenetics (São Paulo, Brazil), and were maintained under standard conditions at the laboratory’s animal facility of the Immunogenetics Laboratory at Butantan Institute (São Paulo, Brazil) (CEUAIB nº 6597290524).

### 2.3. Induction of Acute Inflammation in the AIRmax Model

**Figure 1:**
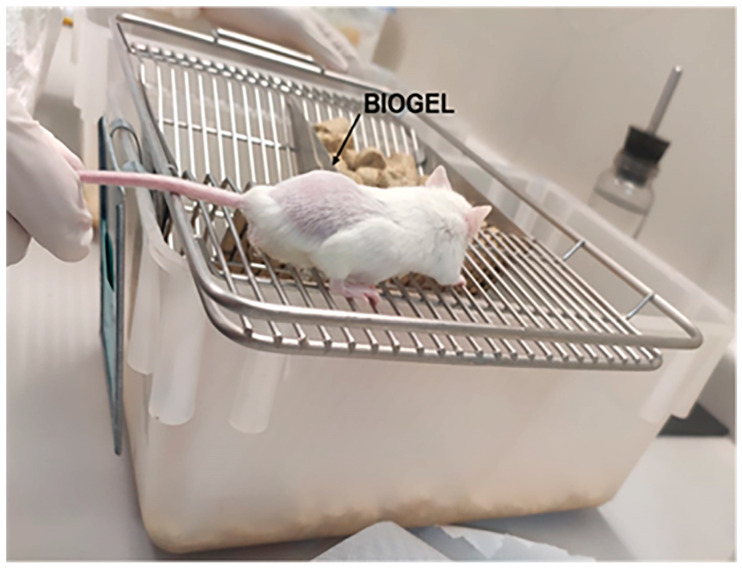
Inducing acute inflammation in the AIRmax model. The photograph illustrates the precise localization of the Biogel P-100 (Biogel P-100, Bio-Rad^®^, Hercules, CA, USA) suspension after administration. A total of 750 µL of the polyacrylamide particle suspension was injected into the subcutaneous tissue of the lumbar region of a previously shaved AIRmax mouse to induce acute inflammation. The image shows the resulting localized swelling at the injection site.

Male and female AIRmax mice aged 8 to 12 weeks, weighing 20 to 25 g, were used (Table 1). These mice were produced and maintained by the Immunogenetics Laboratory of the Butantan Institute [25].To induce acute inflammation, 750 μL of the polyacrylamide particle suspension of a sterile 67% suspension (53 mg dry weight/mL) of Biogel P-100 (Bio-Rad) prepared in phosphate-buffered saline (PBS) was injected subcutaneously (Bio-Rad^®^, Hercules, CA, USA) into the back of previously shaved mice (Figure 1). The experimental procedures were performed following the principles of the ethics committee on the use of animals at the Butantan Institute (CEUAIB nº 6597290524).

### 2.4. Cell Therapy

**Figure 2:**
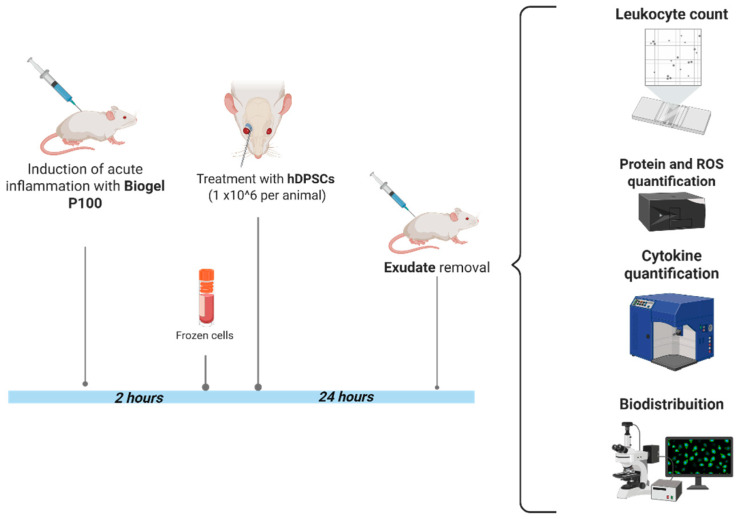
Experimental flowchart and analysis protocol. This flowchart outlines the key steps, from inflammation induction to the final analysis of therapeutic outcomes, in the AIRmax murine model. Induction of acute inflammation: Acute inflammation was induced in the dorsal, shaved region of AIRmax mice by administering 750 µL of Biogel P-100 suspension subcutaneously. Cell therapy (treatment): Two hours (2 h) post-inflammation induction, the mice received the cell therapy: 1 × 10^6^ hDPSCs suspended in 100 µL of saline solution, injected via the retro-orbital venous plexus. Endpoint analysis: After a 24 h hDPSC treatment period, the animals were euthanized, and the inflammatory exudate was collected and aliquoted. Exudate analysis: The collected exudate was used for subsequent measurement of protein, cytokine, and reactive oxygen species (ROS) levels, as well as total and differential cell counts. Biodistribution study: To track the administered cells, in vivo and ex vivo analyses of hDPSC biodistribution were performed (CEUA nº 6597290524).

Just 2 h after the initial Biogel P-100 injection, the time point at which the acute inflammatory response was actively ramping up—the animals received the hDPSC therapy. Each mouse was injected with 1 × 10^6^ hDPSCs suspended in 100 µL of saline solution. This injection was delivered via the retro-orbital venous plexus. To minimize discomfort and adhere to ethical standards, the mice were first given local and topical anesthesia to the eye prior to administration of the cell suspension (Figure 2).

### 2.5. Blood Cell Count Analsys

To ensure the cellular components remained intact, blood samples ranging from 250 to 500 µL were collected using BD Vacutainer^®^ tubes containing the anticoagulant K2EDTA (BD Diagnostics, São Paulo, SP, Brazil). EDTA is essential for preserving the cellular morphology. Immediately following collection, complete blood counts (CBCs) were performed. This analysis was carried out using an automated hematology analyzer (IDEXX ProCyte DX, IDEXX Laboratories) (Table 2).

### 2.6. Inflammatory Exudate

**Figure 3:**
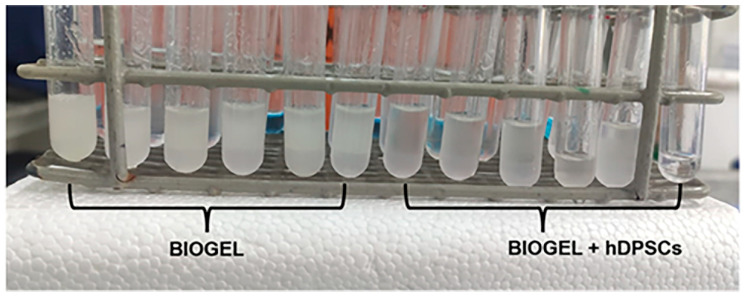
Inflammatory exudate samples post-treatment. This photograph displays inflammatory exudate samples following the experimental protocol. The image is divided into two main groups: the first six tubes, labeled “BIOGEL,” contain exudate from the control animals that did not receive treatment with hDPSCs. The remaining six tubes, labeled “BIOGEL + hDPSCs,” contain samples from the animals that received the hDPSC cell therapy. These tubes represent the raw material used for subsequent cell counting and analysis of inflammatory mediators.

The process for collecting and preparing the inflammatory exudate began 24 h after the cell therapy. To retrieve the exudate from the inflamed site (the subcutaneous tissue of the back), 1 mL of PBS containing 20 U/mL heparin was injected with a syringe directly into the area where the Biogel P-100 had been applied. The resulting fluid was immediately aspirated and transferred to a 15 mL Falcon tube (Figure 3). To separate the large, non-cellular Biogel particles, the tube was left at room temperature for 10 min, allowing the particles to settle at the bottom. Once settled, the supernatant was carefully aspirated and then centrifuged for 5 min at 200× *g* (RCF). Following this spin, the resulting supernatant, which contained the soluble inflammatory mediators, was collected, transferred to a new 500 µL conical-bottom microtube, and promptly stored at −80 °C for later cytokine and protein assays. The pellet, which contained the cells, was resuspended in 500 µL of PBS; this cellular suspension was subsequently used to count neutrophils.

### 2.7. Leukocyte Count in Inflammatory Exudate

For the absolute leukocyte count in the inflammatory exudate, the 500 µL cell suspension was diluted in 2 mL of PBS (1:5 dilution). Next, 100 µL of this diluted cell suspension was transferred to a separate tube containing 400 µL of methylene blue in acetic acid. Turk’s solution was prepared using 1% methylene blue in alcohol and 1% acetic acid. Finally, 30 µL of this treated aliquot was loaded into a Malassez hemocytometer chamber to determine the absolute concentration of cells per unit volume.

### 2.8. Protein Measurement by Absorbance (Preliminary)

Protein concentrations were estimated by measuring absorbance at 280 nm using a microplate spectrophotometer. Samples were diluted in 50 mM phosphate buffer (pH 7.4) and loaded into 24-well plates. Absorbance was recorded directly, and protein levels were expressed in arbitrary units (AU). Values are represented as the mean ± SD of triplicate measurements.

### 2.9. Reactive Oxygen Species (ROS) Detection Assay

Intracellular ROS levels in exudate-derived cells were measured using the fluorescent probe CM-H_2_DCFDA (Thermo Fisher Scientific, Eugene, OR, USA). Cells were centrifuged and resuspended in PBS containing 5 µM CM-H_2_DCFDA, followed by incubation at 37 °C for 30 min. After incubation, cells were washed once with PBS to remove excess probe and resuspended in probe-free PBS. Fluorescence was measured in 24-well plates using a Tecan Infinite M Plex microplate reader (excitation: 485 nm; emission: 530 nm).

### 2.10. Cytokine Assay by Cytometric Bead Array (CBA)

Tumor necrosis factor-alpha (TNF-α) levels in the inflammatory exudate were measured using the murine Th1/Th2/Th17 Cytometric Bead Array (CBA) Kit (BD Biosciences, San Jose, CA, USA) according to the manufacturer’s instructions. Samples were analyzed by flow cytometry using a BD^®^ Canto II instrument (Multiuser facility, Butantan Institute, Brazil).

### 2.11. Protein Quantification by BCA Protein Assay

Total protein concentration was determined using the BCA Protein Assay Kit (Abcam, Cambridge, UK). Briefly, a working reagent was prepared by mixing the BCA reagent with the copper reagent in a 50:1 ratio. This reagent was added to samples and standards in a 96-well plate, followed by incubation at 37 °C for 30 min. Absorbance was measured at 562 nm. Protein concentrations were calculated based on a standard curve generated from bovine serum albumin (BSA) standards (range: 0.5–30 µg). 

### 2.12. In Vivo Biodistribution Assay

To enable bioluminescence imaging (BLI), hDPSCs (passage 2) were genetically modified to express the firefly luciferase gene using RediFect ™ Red-FLuc-Puromycin Lentiviral Particles (PerkinElmer, Waltham, MA, USA).

Briefly, 5 × 10^4^ cells were seeded per well in a 12-well plate. After 12 h, the medium was replaced with Opti-MEM (Gibco) to facilitate transfection. Cells were transduced at a multiplicity of infection (MOI) of 10 or 20. Twenty-four hours post-transduction, cells were washed twice with PBS, and fresh medium containing 0.5 µg/mL Puromycin (Thermo Fisher Scientific) was added for selection. The selection medium was refreshed every 3 days for 10 days.

Successful transfection was confirmed by plating selected cells (5 × 10^3^ to 5 × 10^4^) in a 96-well plate. After 24 h, D-luciferin (XenoLight D-Luciferin Potassium Salt) was added, and bioluminescence was detected using the IVIS^®^ Spectrum In Vivo Imaging System (PerkinElmer). Confirmed FLuc-expressing hDPSCs were expanded until the fifth passage [36].

For the biodistribution assay, animals received an intradermal injection of Biogel in the lumbar region, followed by a systemic administration of 1 × 10^6^ transfected hDPSCs via the retro-orbital sinus. Bioluminescence was monitored at 2, 6, and 24 h post-injection using the IVIS® Spectrum system (PerkinElmer, Waltham, MA, USA).

### 2.13. CellTrace Violet Ex Vivo Tracking

hDPSCs were labeled with CellTrace™ (Invitrogen, Thermo Fisher Scientific), according to the manufacturer’s instructions. Cells were washed with PBS and incubated with the CellTrace solution in serum-free medium for 20 min at 37 °C, protected from light. The reaction was quenched by the addition of complete medium (containing FBS). After washing, 1 × 10^6^ labeled cells were administered to the animal. At 2, 6, and 24 h post-administration, inflammatory exudate was collected and transferred to 24-well plates. Fluorescence was analyzed using the Cytation™ 3 Cell Imaging Multi-Mode Reader (BioTek Instruments, Winooski, VT, USA).

### 2.14. Statistical Analysis

Statistical analysis was performed using GraphPad Prism version 8.0 (GraphPad Software, San Diego, CA, USA). Data were compared using the unpaired Student’s *t*-test. Results were considered statistically significant at *p* < 0.05.

## 3. Results

### 3.1. Kinetics of Inflammatory Response After hDPSC Treatment

**Figure 4:**
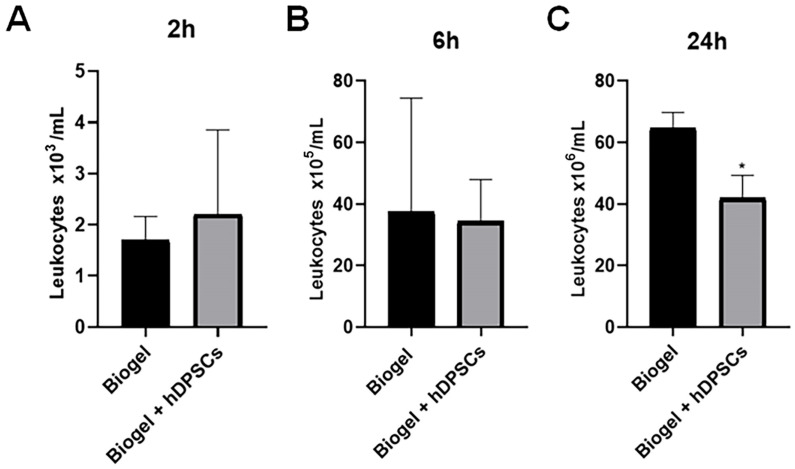
Kinetics of total leukocyte migration into the inflammatory exudate. Total leukocyte counts were quantified in exudates from Biogel-treated animals, with or without hDPSCs, across three time points (2, 6, and 24 h) (**A**–**C**). No significant differences in leukocyte migration were observed at the early time points (2 and 6 h) between the control and hDPSC-treated groups (**A**,**B**). Notably, at the 24 h time point, hDPSC treatment significantly reduced in total leukocyte infiltration compared to the Biogel control (* *p* < 0.05) (**C**). These results indicate delayed but effective immunomodulatory action by the hDPSCs, culminating in reduced cellular recruitment by 24 h. Data are presented as the mean ± SD, and statistical significance was determined using Student’s *t*-test.

To confirm the established AIRmax phenotype (as characterized by [25], we first validated the acute inflammatory response by quantifying leukocyte counts and secreted proteins in the exudate following inflammation induction. To characterize inflammatory kinetics, measurements were taken 2, 6, and 24 h post-induction to determine the optimal window for hDPSC treatment (Figure 4A–C). The time points of 2 and 6 h were selected to capture the early phases of the acute inflammatory response in the AIRmax model. The 24 h time point represents both the peak of acute inflammation and the maximum duration permitted by the institutional ethics committee for maintenance of the Biogel-induced inflammatory stimulus.

#### 3.1.1. Leukocyte Migration

Following treatment with hDPSCs for 2, 6, and 24 h (Figure 4A–C), the inflammatory exudate was harvested to quantify the total number of leucocytes recruited to the site of inflammation. The absolute leukocyte counts across three time points are shown in Figure 4A (2 h) and Figure 4C (24 h).

Analyzing the results presented in Figure 4, we observed that the 2 h hDPSC treatment group showed no significant difference in leukocyte counts compared to the Biogel control group (Figure 4A). Similarly, the 6 h hDPSC treatment group also exhibited no significant difference in leukocyte migration when compared to the control group (Figure 4B). However, a significant reduction (*p* < 0.05) in the number of migrating leukocytes was observed in the exudate 24 h after hDPSC administration (Figure 4C). This finding demonstrates that the hDPSCs are capable of reducing inflammatory leukocyte migration, but this therapeutic effect requires a treatment period of at least 24 h to become statistically significant.

#### 3.1.2. Protein Quantification

**Figure 5:**
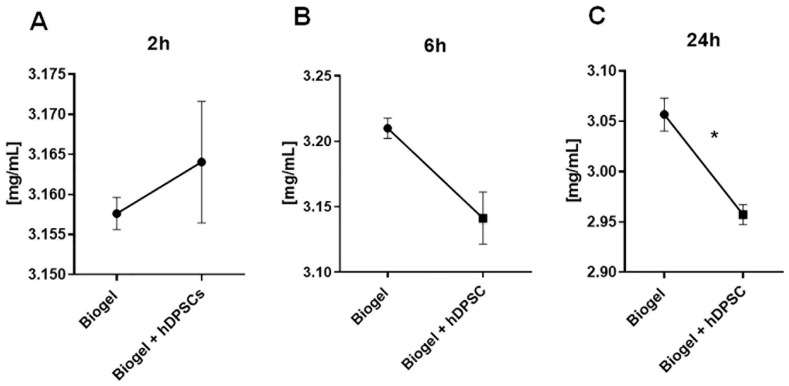
Total protein concentration in inflammatory exudates. This Figure illustrates total protein levels measured in the exudates of Biogel-treated animals, with or without hDPSC, at three different time points. Analysis revealed no significant differences in protein levels at 2 h (**A**) or 6 h (**B**). However, at 24 h (**C**), hDPSC treatment significantly reduced protein concentrations (*p* < 0.05) *, and is indicated by asterisks (*), showing an effective modulation of vascular permeability and protein extravasation by cell therapy. Data is presented as the mean ± SD, and statistical analysis was performed using Student’s *t*-test.

Following the collection of the inflammatory exudate at 2, 6, and 24 h post-hDPSC treatment, the fluid was subjected to a total protein absorbance assay (A280) to quantify secreted protein levels. These proteins serve as critical indicators of vascular permeability and the intensity of the acute inflammatory response. The total protein levels measured in the exudate after 2, 6, and 24 h of treatment are presented in Figure 5A, Figure 5B, and Figure 5C, respectively.

Analysis of the results in Figure 5A revealed no statistical difference in secreted protein levels between the control and hDPSC-treated group at the 2 h mark. This lack of effect persisted at the 6 h time point (5B), where hDPSC administration did not significantly alter protein secretion. However, a significant shift was observed following 24 h of hDPSC treatment (Figure 5C): cell therapy significantly reduced the levels of secreted proteins in treated animals compared to the controls (*p* < 0.05).

This finding mirrors the leukocyte migration data and reinforces that the 24 h interval is the optimal time point for evaluating the therapeutic potential of hDPSCs in this model.

### 3.2. Effects of hDPSC Treatment at the Standardized Time Point

The AIRmax and AIRmin mouse strains are powerful tools for studying inflammation due to their opposing genetically determined inflammatory baselines: AIRmax exhibits a high inflammatory response, while AIRmin shows a low inflammatory response. This unique pairing allows us to test anti-inflammatory therapies, like hDPSCs, under contrasting different underlying inflammatory conditions.

#### 3.2.1. Leukocyte Migration in Inflammatory Exudate After 24 H Treatment

Having established that the 24 h interval produced the most pronounced effects, we standardized this time point. We then expanded the study, increased the sample size, and included AIRmin mice as a comparative phenotype. Leukocyte migration was assessed 24 h post-treatment.

**Figure 6:**
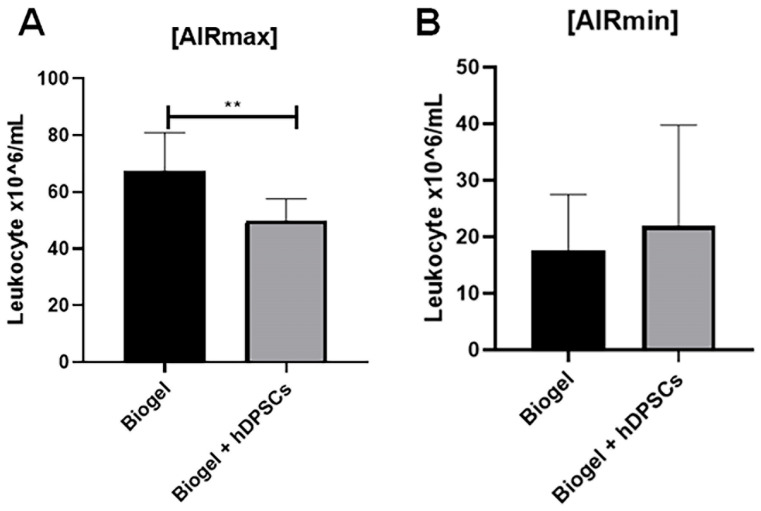
Leukocyte recruitment following 24 h hDPSC treatment in AIRmax and AIRmin mice. This Figure compares the total leukocyte counts in the inflammatory exudates of AIRmax and AIRmin mice 24 h post-treatment. AIRmax animals (**A**): hDPSC treatment resulted in a significant reduction in leukocyte migration (*p* < 0.01) ** compared to the Biogel-only control group, highlighting the anti-inflammatory efficacy of hDPSCs in a high-inflammation environment. AIRmin animals (**B**): No significant difference was observed between the Biogel-only group and the Biogel + hDPSC group, which is consistent with the naturally low baseline inflammatory response of this strain. Data is presented as the mean ± SD, and statistical analysis was performed using Student’s *t*-test. These combined findings suggest that hDPSCs exert immunomodulatory effects by regulating immune cell trafficking to the inflammatory site. Importantly, hDPSC administration did not exacerbate inflammation in the AIRmin model, confirming the safety of the therapy even in a minimal-response setting. Collectively, these results underscore the therapeutic potential of hDPSCs for modulating acute inflammatory responses, particularly in hyper-inflammatory phenotypes.

In the AIRmax group (Figure 6A), animals that received only the Biogel P100 stimulus exhibited a high average leukocyte concentration of 62.20 × 10^6^ cells/mL in the exudate. In sharp contrast, animals treated with hDPSCs for 24 h showed a reduced average concentration of 49.73 × 10^6^ ** cells/mL. This decrease was statistically significant (*p* < 0.01). This demonstrates that hDPSCs effectively attenuate leukocyte recruitment in a high-inflammatory environment.

In the AIRmin animals (Figure 6B), no significant difference in leukocyte migration was observed between the Biogel-only group and the hDPSC-treated group. This result aligns with the naturally low baseline inflammatory response in the AIRmin model, which provides little room for further reduction by an anti-inflammatory therapy.

#### 3.2.2. Protein Analysis After 24 h Treatment

Following leukocyte quantification, the exudate supernatant was analyzed to evaluate protein secretion, a key indicator of vascular permeability and inflammation.

**Figure 7:**
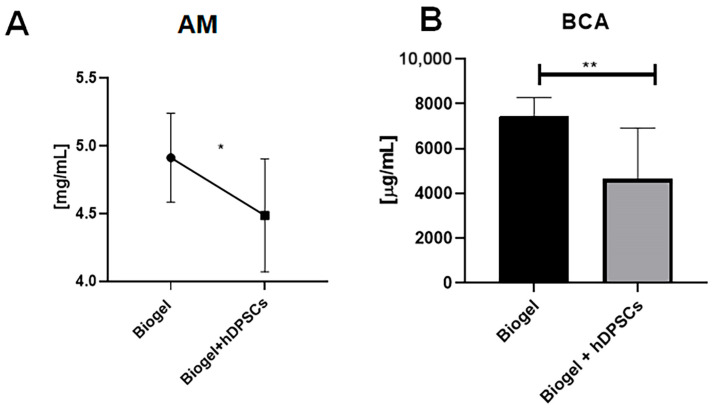
Protein quantification in AIRmax mice (24 h treatment). This Figure presents two complementary assessments of total protein levels in the inflammatory exudates of AIRmax mice 24 h after treatment with Biogel, compared to those treated with Biogel + hDPSCs. (**A**) Absorbance method: Initial analysis revealed that hDPSC treatment significantly reduced (* *p* < 0.05) secreted protein levels compared to the Biogel control. (**B**) BCA assay confirmation: The BCA colorimetric assay confirmed these findings, showing a significant decrease (** *p* < 0.01) in protein concentration following hDPSC therapy.

In AIRmax mice, Figure 7 presents two complementary assessments of protein concentration. Analysis by the absorbance method (Figure 7A) showed that the Biogel-only control group exhibited elevated protein levels. Notably, treatment with hDPSCs resulted in a significant reduction (*) in protein concentration compared to the untreated group, indicating that hDPSCs effectively modulate protein release. This finding was further validated by the BCA colorimetric assay (Figure 7B), which confirmed that protein levels in the Biogel group were significantly higher (*p* < 0.01) ** than those in the Biogel + hDPSCs group. Together, these data support the ability of hDPSCs to attenuate inflammatory protein secretion following Biogel-induced acute inflammation.

**Figure 8:**
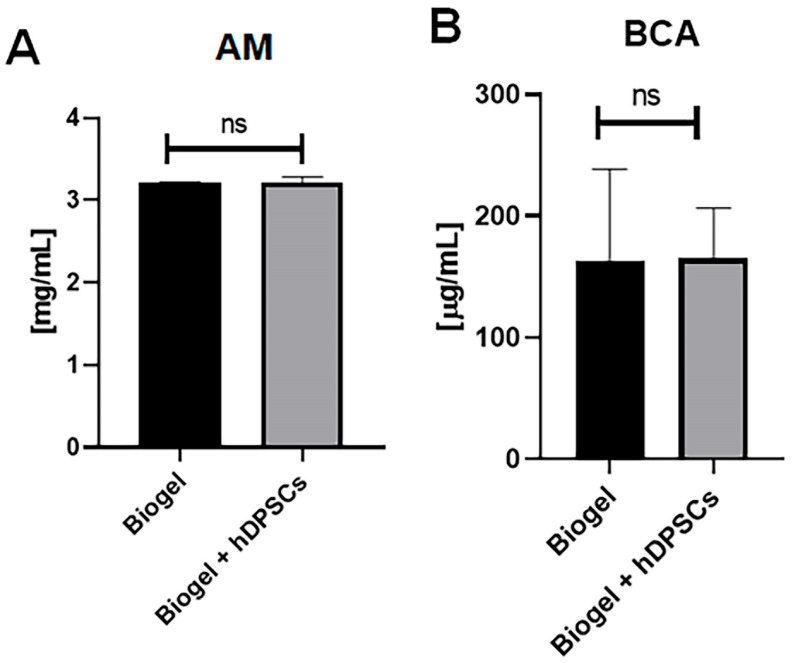
Protein quantification in AIRmin mice (24 h treatment). The total protein levels measured in the inflammatory exudates of AIRmin mice 24 h after treatment with Biogel, compared to those treated with Biogel + hDPSCs. (**A**) Absorbance method: The assay showed no significant (ns) differences in secreted protein levels between the Biogel-only group and the hDPSC-treated group. (**B**) BCA assay confirmation: Similarly, the BCA assay confirmed the absence of significant differences in protein concentration between the two groups. All data are presented as the mean ± SD, and statistical significance was determined using Student’s *t*-test.

In AIRmin mice, protein quantification by the absorbance method (Figure 8A) and the BCA assay (Figure 8B) revealed no significant differences between the Biogel-only and Biogel + hDPSCs groups. This outcome confirms that hDPSC treatment does not alter protein secretion in this naturally low-inflammatory environment.

These results demonstrate that AIRmax animals respond more robustly to hDPSC therapy, exhibiting a pronounced modulatory effect on protein secretion in the inflammatory exudate. Importantly, hDPSC treatment did not increase the production of proteins associated with inflammation, highlighting their potential as a safe and effective strategy to modulate inflammatory responses without exacerbating tissue damage.

#### 3.2.3. Blood Cell Count

**Figure 9:**
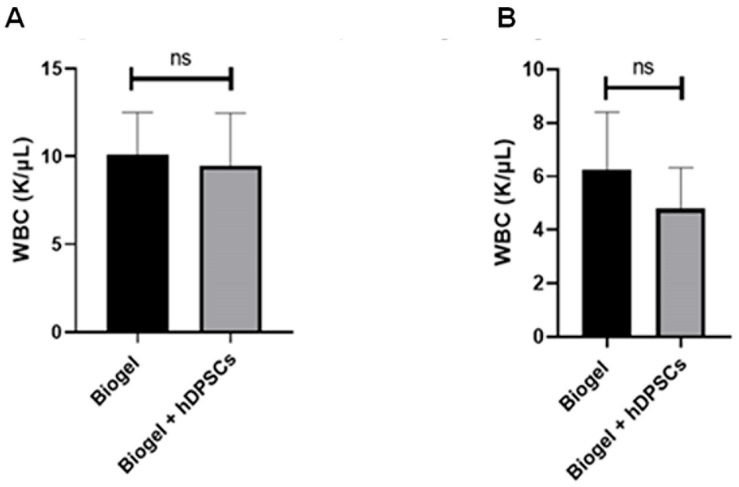
Systemic leukocyte count (WBC) following 24 h treatment. Systemic WBC counts obtained from the hematological analysis of AIRmax (**A**) and AIRmin (**B**) mice 24 h post-treatment. Both AIRmax and AIRmin (**B**) groups showed no significant (ns) differences in circulating WBC counts between the Biogel-only controls and the groups treated with hDPSC. All data are presented as the mean ± SD, and Student’s *t*-test was used for statistical comparison.

To evaluate the systemic influence of hDPSC treatment on the body’s circulating immune cells, we performed a hematological analysis focusing on white blood cell (WBC) counts. The analysis showed that the groups treated with hDPSCs, in both the AIRmax and AIRmin strains (Figure 9A,B), did not result in significant differences in total WBC counts compared to their respective untreated control groups. This demonstrates that the cell therapy did not induce significant alterations in the systemic leukocyte profile.

These findings suggest that the anti-inflammatory and immunomodulatory effects of hDPSCs, while pronounced at the local site of inflammation (exudate), do not perceptibly alter systemic hematological parameters within the bloodstream. Critically, these data corroborate the safety of the hDPSC therapy, as the treatment did not trigger a systemic acute inflammatory response (AIR) or promote an undesirable increase in circulating leukocytes, thereby maintaining hematological homeostasis.

#### 3.2.4. ROS Production After 24 h Treatment

We analyzed reactive oxygen species (ROS) production in leukocytes isolated from the inflammatory exudate to determine if hDPSC treatment modulates oxidative stress during acute inflammation.

**Figure 10:**
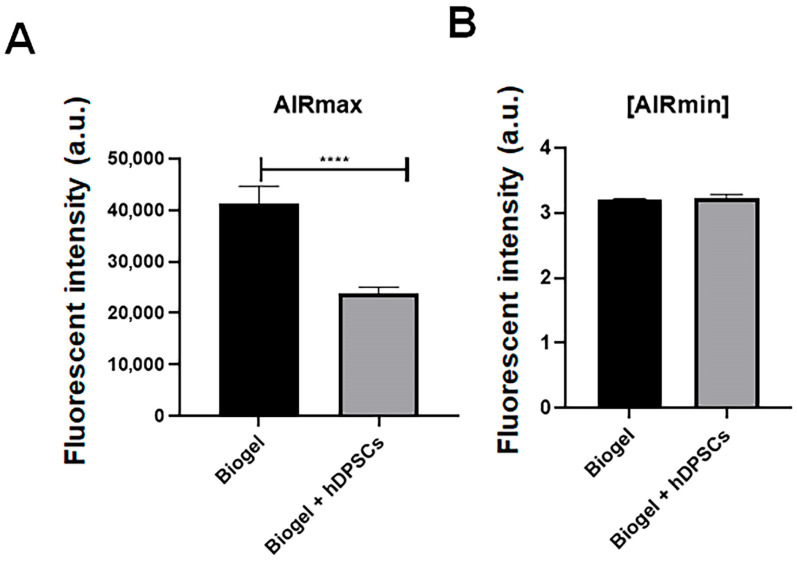
ROS production in inflammatory exudates following 24 h treatment. (**A**) AIRmax animals: hDPSC treatment resulted in a highly significant decrease in ROS levels (*p* < 0.0001) **** compared to the Biogel-only group. This finding indicates that hDPSCs effectively mitigate oxidative stress in a high-inflammatory environment. (**B**) AIRmin animals: No significant (ns) difference in ROS production was observed between the Biogel-only and hDPSC-treated groups, which is consistent with the low baseline inflammatory response and minimal oxidative stress in this model. All data are presented as the mean ± SD, and statistical significance was determined using Student’s *t*-test.

In AIRmax mice (Figure 10A), the 24 h treatment with hDPSCs resulted in a significant reduction in ROS levels compared to the untreated animals (*p* < 0.05). This decrease indicates that hDPSCs effectively mitigate oxidative stress, a key driver of leukocyte-mediated tissue damage and the amplification of the inflammatory response. This reduction suggests that hDPSCs not only limit leukocyte infiltration but also exert cytoprotective effects by modulating intracellular oxidative pathways, underscoring their potential as an anti-inflammatory and cytoprotective therapy in settings of high inflammation.

In AIRmin mice (Figure 10B), these low baseline inflammatory responders exhibited no significant change in ROS production following hDPSC treatment. This finding is consistent with the minimal oxidative stress expected in this model, suggesting that the modulatory effects of hDPSCs on ROS are specifically pronounced under conditions of heightened inflammation. Collectively, these results demonstrate that hDPSCs can attenuate oxidative stress in highly inflamed environments, further reinforcing their therapeutic potential in the management of acute inflammatory responses.

#### 3.2.5. Cytokine Secretion in Exudate After 24 H Treatment

To investigate the effect of hDPSC treatment on the inflammatory milieu, cytokine levels in the exudate supernatants were analyzed using CBA.

**Figure 11:**
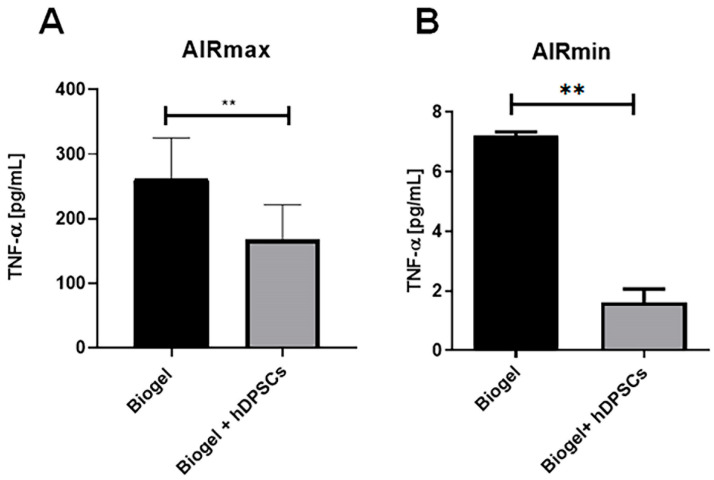
TNF-α quantification following 24 h treatment. (**A**) AIRmax animals: hDPSC treatment resulted in a significant reduction in TNF-alpha levels (*p* < 0.01) ** compared to the Biogel-only control group. (**B**) AIRmin animals: Notably, hDPSC treatment also significantly decreased TNF-alpha levels (*p* < 0.01) ** in this low-inflammatory strain, despite the low baseline. All data are presented as the mean ± SD, and statistical significance was determined using Student’s *t*-test.

In AIRmax mice (Figure 11A), the 24 h hDPSC treatment resulted in a significant reduction (**) in TNF-alpha levels compared to untreated animals. This clearly indicates that hDPSCs exert a potent anti-inflammatory effect by suppressing a key pro-inflammatory mediator involved in acute inflammation. This finding complements the previously observed decreases in leukocyte migration and ROS production, highlighting the ability of hDPSCs to modulate multiple cellular and molecular aspects of the inflammatory response in a high-inflammatory environment.

In AIRmin mice (Figure 11B), hDPSC treatment also significantly reduced TNF-alpha levels, despite the lack of significant effects on leukocyte recruitment or ROS production in this strain. This is a crucial distinction, suggesting that hDPSCs can directly modulate cytokine secretion at the molecular level, independently of cellular infiltration or oxidative stress. This demonstrates a robust immunomodulatory capacity across different inflammatory phenotypes.

Collectively, these observations clearly highlight that hDPSCs can modulate both the cellular and molecular components of acute inflammation. These results further support the therapeutic potential of hDPSCs in phenotypes characterized by heightened inflammatory responses, while simultaneously confirming their safety as they do not exacerbate inflammation in low-responder contexts.

#### 3.2.6. In Vivo Biodistribution of hDPSCs (BLM Assay)

To track the hDPSCs following systemic injections, cells were transfected with the luciferase gene, enabling longitudinal evaluation via bioluminescence imaging (BLI). Following the induction of acute inflammation with Biogel P100, animals received a retro-orbital injection of 1 × 10^6^ hDPSCs. Imaging was performed at 2, 6, and 24 h post-injection.

**Figure 12:**
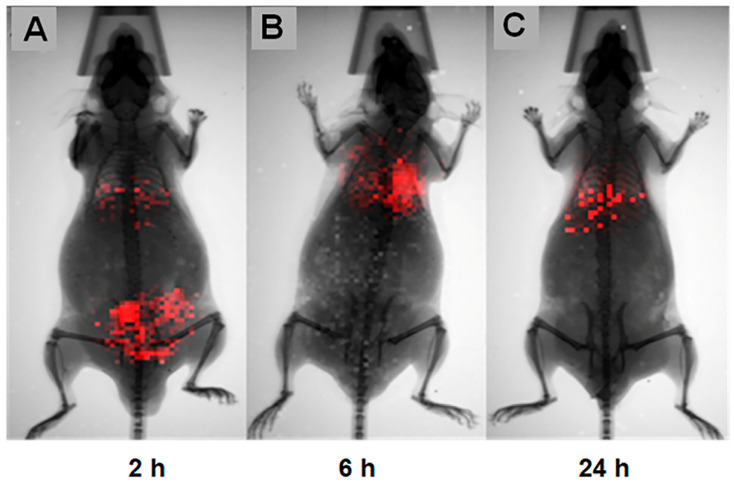
In vivo biodistribution of luciferase-transfected hDPSCs. This Figure illustrates the longitudinal tracking of hDPSCs using BLI. (**A**) 2 h post-treatment: A prominent bioluminescent signal indicates rapid hDPSC distribution. Signals are clearly visible at the site of acute inflammation (lumbar region), as well as within the thoracic region. (**B**) 6 h post-treatment: The signal at the localized inflammation site is absent or below the detection threshold, while the bioluminescence remains concentrated in the thoracic region, indicating rapid clearance from the subcutaneous site. (**C**) 24 h post-treatment: The signals persist in the thoracic region, albeit with a decreased intensity compared to the 6 h time point.

Two hours post-injection (Figure 12A), the hDPSCs were distributed systemically. Notably, a distinct signal was detected in the lumbar region coinciding with the Biogel application site, confirming that the hDPSCs successfully homed to the site of acute inflammation in the AIRmax mice. Furthermore, a robust bioluminescent signal was observed in the thoracic region, corresponding to pulmonary cell entrapment.

Six-hour post-injection (Figure 12B), the signal in the lumbar region (Biogel site) was no longer detectable. Meanwhile, the concentration in the pulmonary region (thoracic) had noticeably increased.

Twenty-two hours post-injection (Figure 12C), the bioluminescent signal remained present in the lung region, albeit with decreased intensity, up until 24 h after cell therapy. These results confirm that hDPSCs initially home to the primary inflammation site.

#### 3.2.7. Biodistribution Analysis by Fluorescence In Vitro

**Figure 13:**
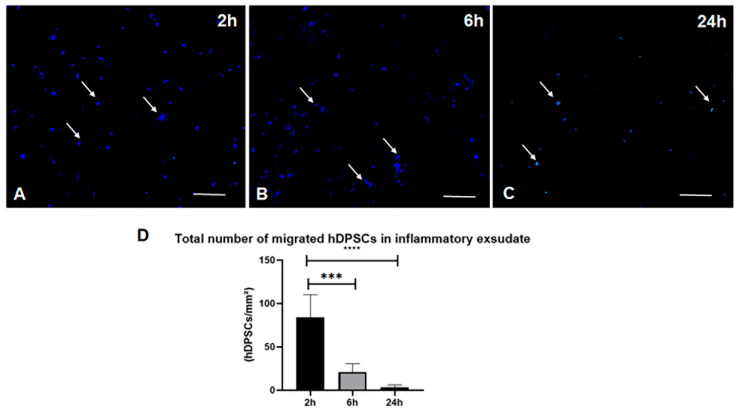
Representative immunofluorescence photomicrographs of inflammatory exudates. Exudates were collected at 2 h, 6 h, and 24 h following the administration of hDPSCs labeled with CellTrace Violet (Pacific Blue channel). (**A**) A higher density of positive cells is observed at 2 h post-treatment. (**B**) A reduction in cell frequency is visible at 6 h, followed by (**C**) a significant decrease by the 24 h mark (***). (**D**) The accompanying graph displays the quantification of positive hDPSCs performed using ImageJ software (1.54p Version). Data are presented as mean ± SD. Statistical significance: 2 h vs. 6 h (****, *p* < 0.0001) and 2 h vs. 24 h (***, *p* < 0.001). White arrows indicate cells positive for CTV staining for better visualization. Scale bar = 50 µm.

To confirm the homing effect of hDPSCs to the site of inflammation, an ex vivo tracking assay was performed using cells labeled with the fluorescent marker CellTrace Violet (CTV). The inflammatory exudate was collected at the same critical time points used in the previous bioluminescence assay: 2 h, 6 h, and 24 h post-treatment. Figure 13 shows representative images of the inflammatory exudate collected at these time points, illustrating the temporal dynamics of hDPSC migration.

Two hours post-treatment (Figure 13A): This image shows a high density of CTV-labeled cells. This finding is consistent with the early BLI results, confirming that a significant number of hDPSCs successfully migrated to the inflammation site within the first few hours of treatment.

Six-hour post-treatment (Figure 13B), the CTV-labeled cells were more dispersed and at a lower density. While hDPSCs were still present, their quantity was noticeably reduced compared to the 2 h sample.

Twenty-four hours post-treatment (Figure 13C), the fluorescence intensity was considerably lower, with only a few positively labeled cells detected in the exudate.

Collectively, these ex vivo results corroborate the in vivo BLI findings: hDPSCs achieve rapid homing to the inflamed site; however, their presence in the localized exudate is transient, with a significant decrease in cell numbers observed between 6 and 24 h.

#### 3.2.8. Quantitative Analysis of hDPSC Homing Dynamics

To complete the qualitative immunofluorescence findings, image quantification was performed using Image software to determine the average number of hDPSCs present in the exudate at each experimental time point (2 h, 6 h, and 24 h) (Figure 13D). The resulting quantitative data, analyzed using GraphPad Prism, demonstrated a progressive and statistically significant reduction in the number of hDPSCs detected in the exudate over the 24 h treatment period.

Statistical analysis revealed a significant difference (**** *p* < 0.0001) between the 2 h and 6 h time points, and a significant decrease (*** *p* < 0.001) was observed when comparing the 2 h and 24 h time points. These findings indicate a clear temporal decline in hDPSC counts at the site of inflammation, quantitatively corroborating the visual observation of the representative images. These data confirm that while hDPSCs rapidly home to the inflammatory site, they are subsequently cleared or redistributed to other organs, such as the lungs, as previously indicated by the BLI data.

## 4. Discussion

Our results consistently demonstrate the potent therapeutic capabilities of hDPSCs in modulating acute inflammation. The significant decrease observed in the total leukocyte count within the inflammatory exudate after hDPSC treatment supports the hypothesis that this cell therapy acts by suppressing local leukocyte recruitment [37,38]. Additionally, the parallel reduction in protein extravasation can be attributed to the well-known immunomodulatory and anti-inflammatory potential of MSC, which is consistent with an indirect stabilization of the endothelial barrier. This suggests that hDPSC therapy effectively modulates vascular permeability and the leakage of plasma proteins into the interstitial space [39,40].

Given that inflammatory mediators usually promote increased endothelial permeability—the primary driver of protein migration [41]—our data suggest that lower protein concentration reflects reduced endothelial activation and a more regulated inflammatory environment, thereby preventing excessive tissue damage.

A central molecular finding was the significant reduction in TNF-α levels in the exudate of treated animals. TNF-α is central to the initiation of acute inflammation, driving both leukocyte migration and vascular permeability [42,43]. By attenuating this primary pro-inflammatory cytokine, hDPSCs likely prevent the progression from acute to chronic inflammation [44].

Interestingly, in the low-inflammatory AIRmin mice, TNF-alpha levels significantly decreased despite the absence of significant changes in leukocyte migration or ROS production. This suggests a direct molecular modulatory effect on cytokine production that operates independently of cellular infiltration or oxidative stress. These findings align with previous reports in the literature on the inherent immunomodulatory capacity of hDPSCs, including the suppression of TNF-alpha and enhancement of anti-inflammatory mediators like IL-10 [45,46,47]. Furthermore, the greater efficacy observed in AIRmax mice corroborates evidence that the anti-inflammatory effects of hDPSCs are more pronounced in highly inflamed microenvironments [48,49].

Additionally, hDPSC treatment significantly reduced the levels of ROS. ROS are critical mediators in the inflammatory cascade, capable of activating signaling pathways that lead to further production of pro-inflammatory cytokines, like TNF-alpha [15,50,51]. By decreasing ROS, hDPSCs mitigate oxidative stress, promoting inflammation resolution and protecting tissue from oxidative damage.

Our biodistribution data, analyzed via both BLI and CellTrace Violet tracking, presents a compelling and somewhat unconventional view of hDPSC homing kinetics. The general consensus in MSC research is that after intravenous injection, the majority, 80–90%, of cells become mechanically trapped in the pulmonary vasculature (the “first-pass effect”). Only a small fraction is thought to escape this pulmonary trap and successfully home to distant inflamed or injured sites [51]. However, our BLI results at 2 h post-injection show a distinct pattern: a prominent bioluminescent signal was observed at the acute inflammation site (lumbar region), alongside the weak signal in the thoracic region. This indicates a rapid and effective initial homing in on the inflammation site that appears to challenge the absolute dominance of the pulmonary trap observed in other models [52].

While the hDPSCs successfully reached the inflammation site early on, the subsequent tracking revealed their transient presence. By 6 h, the signal in the inflammation site was essentially undetectable, while the signal in the thoracic/lung region increased and persisted until 24 h. This suggests two possibilities:

1. Efficient local clearance: The microenvironment of the subcutaneous Biogel lesion, a non-immunogenic but highly phlogistic (inflammation-inducing) environment, may drive the rapid clearance of hDPSCs after their initial arrival and interaction.
2. Secondary redistribution: The cells detected in the lung at 6 and 24 h may represent two populations: (a) the classic population that was initially mechanically trapped, and (b) the cells that initially homed to the inflammation site but were then rapidly cleared back into the systemic circulation and subsequently trapped or filtered by the lungs. This rapid clearance from the inflammation site is a critical mechanistic finding, highlighting that the presence of hDPSCs at the inflammation focus is transient but functionally sufficient.

The temporal dissociation between peak hDPSC presence and the onset of anti-inflammatory effects supports a paracrine, inflammation-responsive mechanism of action. hDPSCs rapidly sense local inflammatory cues such as TNF-α and oxidative stress, triggering the release of a transient but biologically potent secretome that subsequently reprograms host immune responses. This results in delayed yet sustained suppression of inflammatory mediators, which is consistent with a ‘hit-and-run’ immunomodulatory model.

These secreted factors then engage the host’s own cells to start the healing cascade, a process that requires the full 24 h to fully manifest as reduced leukocyte migration and protein extravasation. While our data strongly support this paracrine mechanism, the precise molecular mediators and direct mechanistic links between hDPSCs and host immune cells remain to be fully elucidated.

In essence, our data confirms that for hDPSCs, speed of homing is vital, but sustained presence is not required; their function is performed rapidly, leaving the host system to carry out the long-term resolution.

## 5. Conclusions

**Figure 14:**
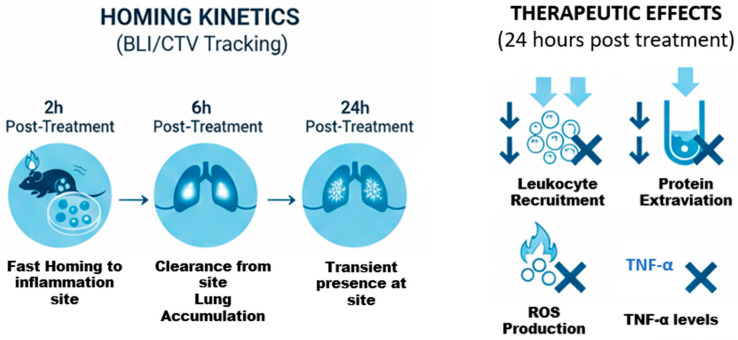
Biodistribution kinetics and therapeutic efficacy of treatment. The (**left panel**) illustrates the homing kinetics via BLI/CTV tracking, showing rapid localization to the lungs and inflammatory site at 2 h, followed by lung accumulation at 6 h and transient site presence at 24 h. The (**right panel**) summarizes the therapeutic effects 24 h post-treatment, highlighting significant reductions in leukocyte recruitment, protein extravasation, ROS production, and TNF-alpha levels, promoting host resolution of inflammation.

Our research utilizing the AIRmax model provides robust evidence of the therapeutic potential of hDPSCs in modulating acute inflammation. The key innovative findings of this study demonstrate that hDPSC treatment significantly attenuates leukocyte recruitment and decreases inflammatory protein secretion in the exudate of the highly inflamed AIRmax mice, indicating a profound anti-inflammatory effect on both cellular infiltration and vascular permeability (Figure 14).

Mechanistically, we confirmed that hDPSCs simultaneously target multiple inflammatory pathways by significantly reducing both ROS production (mitigating oxidative stress) and suppressing levels of the primary pro-inflammatory cytokine, TNF-alpha. Furthermore, while biodistribution analysis confirmed rapid hDPSC homing to the site of inflammation within the first two hours, the most pronounced anti-inflammatory functional benefits only became significant after 24 h (Figure 14).

Therapeutic success is related not merely to rapid homing, but to a brief and critical window of paracrine signaling within the inflamed microenvironment; prolonged cellular persistence is not required for sustained anti-inflammatory effects. These properties—encompassing protection against oxidative damage, reduction in protein leakage, and potent immunomodulation—position hDPSCs as a promising candidate for pathologies defined by heightened inflammatory states, such as neuroinflammatory diseases.

## Figures and Tables

**Table 1:** Experimental groups of animals used in this study.

Animals	Biogel P100 + hDPSCs	Biogel P100
AIRmax	n= 31	n = 31
AIRmin	n= 12	n =12

**Table 2:** Established reference values for complete blood counts (red blood, white blood, and platelet counts) for all mouse strains [35].

Erythroid Series	Leukocyte Series	Platelet Series
RBC—7–11 × 10^6^/µL	WBC—2–10 × 10^6^/µL	PLT—900–1600 × 10^3^/µL
HCT—40–50%	LINF—70–80%	
HGB—10–17 g/dL	GRAN—20–30%	
MCV—13–17 fL	MON—0–2%	
MCH—13–17 pg/mL	EOSIN—0–7%	
MCHC—30–38 g/dL	BASO—0–1%	

## Data Availability

The data used and/or analyzed during the current study are available from the corresponding author on reasonable request.
